# The Attitude of the Farmer toward the Tuberculin Test1Presented at the Thirty-eighth Annual Convention of the American Veterinary Medical Association, Atlantic City, N. J., September, 1901.

**Published:** 1902-02

**Authors:** Carl W. Gay

**Affiliations:** Ames, Iowa


### THE ATTITUDE OF THE FARMER TOWARD THE TUBERCULIN TEST.[1]

By Carl W. Gay, D.V.M.,
AMES, IOWA.

The subject of this paper is broad, and one upon which considerable has been written; but it is not my intention to enter upon a discussion of the arguments pro and con regarding the tuberculin test. The idea is simply to impress more forcibly the importance of the proper education of the farmer as a means toward eradicating tuberculosis from dairy herds, and to call particular attention to the responsibility of the veterinarian as a factor in the accomplishment of this end. In a paper read before the last annual meeting of the Interstate Association Live-stock Sanitary Boards, Dr. Dalrymple has so cleverly worded the thought which I wish to express that I quote him here, viz.:

"I think that it may be truthfully asserted that the success of all great reforms is largely dependent upon the education of public opinion, ignorance being the mighty retarding factor to the advancement of all progressive movements.

"Scientific progress has been but slow, since the struggle has been for ages against the enormous odds of incredulity, indifference, and superstition. Truth, however, must prevail; and scientific investigation, which is but the searching after truth, although hampered on all sides by those untoward influences which are born of ignorance, superstition, and doubt, is slowly but surely revealing the true nature of things, and must eventually and effectually succeed. Still the rate of progress in the accomplishments of the desired ends will greatly depend upon the acceptance of the revealed truths by the people, which can be satisfactorily attained only through an educated public opinion."

While all the efforts of veterinary sanitarians have been influenced to a greater or less degree by these conditions of incredulity, indifference, and superstition, the opposition seems to have been

[1] Presented at the Thirty-eighth Annual Convention of the American Veterinary Medical Association, Atlantic City, N. J., September, 1901.

directed more particularly at their efforts to eradicate tuberculosis from dairy herds; hence, I believe a brief consideration of some of the reasons for this opposition and our ability to overcome it to be in order at this meeting. Some experience in the practical application of the test by direction of the New York State Board of Health has given me occasion to appreciate, sometimes quite keenly, the attitude of the majority of the farmers toward this work. Inasmuch as all classes of farmers, from the city individual who maintains his country-place simply as a source of recreation to the poor tenant farmer who even rents his cows, were dealt with, their opinions may be taken as representing the position of the farmers generally on this matter.

It is not for me to outline or recommend any course to be adopted as a means of altering the present condition of things, but I venture a few suggestions for your discussion.

To eventually exterminate tuberculosis in cattle from any of our States without the hearty co-operation of the farmer-owners is a task the enormity of which can only be realized by those who have come in intimate contact with the opposing forces. To secure their co-operation we must gain their confidence. To do this it is necessary to overcome their prejudice, and the easiest way to accomplish this is by rational, conservative methods, and, above all, to instruct them in the nature of the disease, its cause, and lesions. A clear explanation of how the disease is transmitted from animal to animal, perpetuated in stables, and the channels through which it is liable to be introduced into sound herds and non-infected premises, will do much toward bringing the owner to see the necessity of such measures as are proposed for its control. To direct all our efforts toward the simple destruction of all tuberculous animals in a given district is like treating disease without removing the cause; and its effect is just as transient. If, however, while we are doing away with the diseased cattle we are posting the owners regarding our work and its relation to the live-stock interest of the commonwealth as well as to the public health, we have established a permanent vigilance committee, in whose presence it will be exceedingly difficult for the disease again to get the upper hand, and by whose efforts its ultimate extinction is sure to come.

To consider briefly the reason for this opposition on the part of the farmers: It is easy for us to understand why a man objects to having his cattle, which he has raised and possibly prizes highly, and upon which he is dependent for a livelihood, condemned as diseased and destroyed when they are apparently in good health and physical condition; and it is natural that these owners, in protection of their own interests, should consider and act upon the statements published in the various agricultural papers and purporting to be reliable and authoritative. Nevertheless, it is highly amusing to note how readily a theory in one of our agricultural journals—which, by the way, seem eager enough to give publicity to these oftentimes sensational statements—is accepted as authoritative. They have more weight with the majority of farmers than the very conclusive evidence of modern research can be made to have on this question. It would seem that men of no professional or scientific prestige whatever have only to advance some theory in opposition to modern views regarding tuberculosis in cattle to spring at once into prominence and be quoted as an authority by cattlemen far and wide. Not long since I had a talk with a member of the firm which a few years ago was foremost among the breeders of Holstein cattle in this country, having owned Pauline Paul, the champion milk and butter cow of the world. To-day this firm is out of business, and it has been intimated that the dispersal of their cattle was due to the existence of tuberculosis in their herd. Whether that was the case or not I could not say, but the disease was known to be present among their animals. As illustrative of the views held by many men prominent in cattle affairs, and who should know better, I will repeat in substance what he said. He was bitterly opposed to the work, and pronounced it as all wrong. He did not propose to have cattle representing hundreds of dollars to him condemned by a test that was not infallible, and be post-mortemed to have only the minutest kernel held up by the veterinarian in charge as the only evidence of the existence of the disease and responsible for the reaction. Furthermore, he believed the test harmful to healthy cattle. He had asked a local physician who had been active in the State control of tuberculosis in cattle if he would care to subject his child to an injection of tuberculin. The doctor replied that he did not know that he would care to inject his child, whereupon the gentleman in question declared that he would not subject his cattle to a treatment which the M.D. did not deem advisable to give to his child. In speaking of another firm in the same city who are getting a fancy price for their milk on account of their cattle having been tuberculin tested and the milk handled in a hygienic manner, he said he not only condemned their policy, but absolutely refused to allow their milk to be used in his family, as coming from cows having been injected with tuberculin. These remarks from an intelligent man—a college graduate and an agriculturist of the first order—would obviously work havoc among the cattle breeders of the country should he choose to make them public, as perhaps he has. It would seem that he is laboring under a delusion, not having seen things in their true light. Did he and his colleagues comprehend the true state of affairs as we know them to exist, they must be open to conviction. Could they be made to associate the condition in which the only lesions were primary foci in glands appearing simply as minute nodules with the other extreme, in which the lungs and liver are a mass of abscesses, perhaps, and the pleuræ, peritoneum, and viscera studded with countless masses of well-formed tubercles, their opinions would undoubtedly be altered. The impunity with which healthy animals may be injected with tuberculin, as demonstrated time and again by careful and complete experiments, should relieve their anxiety in this respect.

It is a deplorable fact that many of the statements responsible for the present attitude of the stock-owners toward the tuberculin test have come from men claiming allegiance to our profession. If they are true to their convictions, as we hope they are, a little educational work would be applicable to them; but if, as in some cases I fear, they are soliciting popular favor and increased sales of certain proprietary remedies the case is the more to be deplored.

The tuberculin test has been condemned and resolutions against it adopted by the National Shorthorn Breeders' Association. This will prove a formidable obstacle to the work of eliminating this disease from cattle.

But the latest, and probably the severest blow we as sanitarians have received is the paper read by Dr. Koch before the British Congress of Tuberculosis. Koch is very positive in his claim that intertransmissibility from cattle to man is impossible, although so far as we know his investigations have only verified those of Dr. Theobald Smith, of Harvard, who showed that bovine and human tubercle bacilli have different morphological and cultural characters and differ in their pathogenesis to experimental animals. However, we may expect to be brought up against that statement at every turn for the next decade. People will not stop to consider that the presiding officer, as soon as Dr. Koch was seated, cautioned those present to be very conservative in their acceptance of Koch's theory; also that those in attendance at the meeting and the medical profession generally were almost as a unit in refusing to accept the theory of this one man as contradicting their own experience and investigations of many eminent scientists the world over. The fact that while pulmonary tuberculosis in human beings has decreased nearly 50 per cent. in the last half-century, intestinal tuberculosis in infants under one year has increased 27 per cent., is significant. Every physician has encountered cases of tubercular meningitis and enteritis in infants on milk diet, furnishing the strongest circumstantial evidence of the transmissibility from cattle to man. We must remember also that Koch is a laboratory worker and in no sense a clinician. It is only a few years since this same man startled the scientific world by claiming to have discovered a therapeutic agent in tuberculin, which we know to-day only as a diagnostic. It is with all due respect to this truly great investigator that we say he has erred once, and we trust time will disprove his latest theory, as it did his claim for tuberculin. But the harm has been done; Koch's words have been widely circulated and enthusiastically received by those eager for some new barrier to be placed in the way of modern sanitary reform. It remains for us to be prepared to meet the opposition thus reinforced as best we may.

I have already tried to show that much of the influence responsible for the present antagonistic attitude of the farmers toward this test has been exerted by agricultural papers and those regarded as authority in stock affairs. Theoretically, perhaps, our first efforts at reconciliation should be directed toward these sources; but practically we can do infinite good among the farmers themselves. First and foremost I would advocate, as I have said before, having all owners present at post-mortems, and giving them as good a demonstration of the lesions as possible. I believe great results can be derived from advertising and holding public post-mortems when it can be done without affecting the interest of the owners. True, you may encounter cases in which the lesions are such that it would be difficult to make the owner believe that anything is wrong; but there are usually others in which the lesions are so marked as to call forth exclamations of disgust and approval of the work from the bystanders; then is the time to point out how readily the disease may advance from the first stage to the last without, in many cases, any physical symptoms—how animals harboring the disease in an occult form may transmit it to other more susceptible animals in the acute form, and so be a source of infection for the whole herd. There is a prevailing opinion among the laity that an animal cannot be tuberculous and possess sound lungs; hence it is advisable before opening a carcass to describe all the lesions which are characteristic, and then give your audience to understand that you may find any or all of them present. Do not let them entertain the notion that the little greenish nodules of the œsophagostomun columbianum are tuberculous ulcers of the intestine, or they will insist that there is nothing in it, as nearly every animal killed possesses these nodules.

It strikes me that, when possible, the destruction of the condemned animals should be at some abattoir or under such conditions that if the lesions were found localized, and not injurious to the food value of the carcass, it might be allowed to go on the market, as is done by the Federal inspectors under direction of the Bureau of Animal Industry. In this way the State would be saved an indemnity, and the owner would get better value for his cattle. This procedure would undoubtedly meet with favor from the owners, and could in no way affect the results we are striving to attain. At any rate, it is most essential that the owner should be present at the killing, and see just what condition his cattle are in when he had believed them sound; seeing is believing, and it is especially true in this instance. People will read about these diseased conditions and look at highly colored diagrams, but nothing has as much weight with them as a good look at the real thing. I have a case in mind where a number of herds in one locality were tested and a large per cent. condemned. The owners were of the very poorest class of tenant farmers, and to say that they were opposed to the test when first approached regarding it would be putting it mildly. They objected so strongly as to threaten violence. The condemned cattle were killed and examined before quite an aggregation of owners, neighbors, and villagers, and no sooner had the first carcass been opened than they were unanimous in expressing satisfaction in the work. While they had demurred at first, they were now enthusiastic. If you were to visit that locality to-day you would find the people better posted on tuberculosis in cattle than many persons who profess to be veterinarians, and they are enthusiastic over the State control work. They will not buy cows unless they can do so subject to the test; they have found a better market for their dairy products since their herds have a clean bill of health, and a decided advantage over their neighbors who have not had their cattle tested. One man who had been most active in opposing the work informed me that the sentiment in the village nearest by had become so strong since my first visit some months before that people came five miles from his place to buy milk for church sociables, etc., because they knew the cows he had left were sound. This fact was very gratifying to him and to myself. This same man came to me with his brother and most sincerely apologized for some remarks they had made regarding this work upon my first visit. They assured me that they had objected upon general principles, as they had been misinformed, but realized now that we were in the right, and they only hoped that the good work would go on. I might cite several other instances similar to this as demonstrating what may be done in an educational way at the same time we are destroying the diseased animals. The influence of a number of such incidents as this must mean a great deal not alone as a local benefit, but as a power for good in our Legislatures.

Another phase of this work which I believe should be laid before the farmers for their consideration is the economic importance of tuberculosis in cattle. Even though Koch's latest theory be accepted universally, and the relation of tuberculosis in cattle to public health completely ignored, we have a problem in economics worthy of all the attention this work now receives. Here, again, we have this same incredulity, indifference, and superstition blinding the farmers to the fact that they are abusing their own interests by hushing this thing up and allowing the disease to gnaw at the vitals of their herds without raising a finger to check it. For instance, I condemned and killed fifteen out of twenty-one head in one of the richest dairy districts in New York State, and that owner told me he had been losing a cow or two every year from tuberculosis, having turned them off for bologna stock for whatever he could get out of them. This farmer said he had known the disease to be prevalent in his locality for years. He was sure that it existed in his son's herd, near by, and said that one of his neighbors every year had drawn two or three head of tuberculous animals back on a hill on his place, until at present this hill was strewn with skeletons and carcasses of animals, representing a large amount of money; still these men could not be prevailed upon to take any measures to exterminate a disease which was every year costing them a large percentage of the proceeds of their dairies.

It may seem superfluous to mention, but it is nevertheless essential, that we as operators in the application of this tuberculin test should be exceedingly careful of our technique and manipulations. Abscesses resulting from infection at the time of injection, or any of the sequelæ which may follow careless methods, are charged by the owners to the evils of tuberculin on healthy animals, and are to be carefully avoided.

From a review of the different methods in vogue in the different countries and States for the eradication of tuberculosis, this one fact is indorsed, viz.: In the country or State having obtained the greatest results in this line—as, perhaps, Denmark, for instance—we find the stock-owners themselves in possession of a good, practical understanding of the disease and the measures taken to oppose it. They have consequently indorsed and supported the Federal and municipal authorities in their endeavors to stamp it out.

In conclusion, I would emphasize that the ultimate extermination of tuberculosis in dairy cattle is to be attained, first, by proper disposal of all tuberculous animals—either by slaughter or quarantine—and, second, by a properly educated public opinion regarding this work.

